# Adjunct n-3 Long-Chain Polyunsaturated Fatty Acid Treatment in Tuberculosis Reduces Inflammation and Improves Anemia of Infection More in C3HeB/FeJ Mice With Low n-3 Fatty Acid Status Than Sufficient n-3 Fatty Acid Status

**DOI:** 10.3389/fnut.2021.695452

**Published:** 2021-08-24

**Authors:** Frank E. A. Hayford, Robin C. Dolman, Mumin Ozturk, Arista Nienaber, Cristian Ricci, Du Toit Loots, Frank Brombacher, Renée Blaauw, Cornelius M. Smuts, Suraj P. Parihar, Linda Malan

**Affiliations:** ^1^Centre of Excellence for Nutrition, North-West University, Potchefstroom, South Africa; ^2^Department of Dietetics, School of Biomedical and Allied Health Sciences, College of Health Sciences, University of Ghana, Accra, Ghana; ^3^International Centre for Genetic Engineering and Biotechnology (ICGEB), Cape Town-Component, University of Cape Town, Cape Town, South Africa; ^4^Institute of Infectious Diseases and Molecular Medicine (IDM), Division of Immunology and South African Medical Research Council (SAMRC) Immunology of Infectious Diseases, University of Cape Town, Cape Town, South Africa; ^5^Pediatric Epidemiology, Department of Pediatrics, Medical Faculty, Leipzig University, Leipzig, Germany; ^6^Laboratory of Infectious Disease Metabolomics, Human Metabolomics, North-West University, Potchefstroom, South Africa; ^7^Wellcome Centre for Infectious Diseases Research in Africa (CIDRI-Africa) and Institute of Infectious Diseases and Molecular Medicine (IDM), University of Cape Town, Cape Town, South Africa; ^8^Division of Human Nutrition, Stellenbosch University, Cape Town, South Africa; ^9^Division of Medical Microbiology, Institute of Infectious Diseases and Molecular Medicine (IDM), Department of Pathology, Faculty of Health Sciences, University of Cape Town, Cape Town, South Africa

**Keywords:** n-3 LCPUFA, fatty acid status, adjunct therapy, C3HeB/FeJ TB model, tuberculosis, immuno-nutrition

## Abstract

Populations at risk for tuberculosis (TB) may have a low n-3 polyunsaturated fatty acid (PUFA) status. Our research previously showed that post-infection supplementation of n-3 long-chain PUFA (LCPUFA) in TB without TB medication was beneficial in n-3 PUFA sufficient but not in low-status C3HeB/FeJ mice. In this study, we investigated the effect of n-3 LCPUFA adjunct to TB medication in TB mice with a low compared to a sufficient n-3 PUFA status. Mice were conditioned on an n-3 PUFA-deficient (n-3FAD) or n-3 PUFA-sufficient (n-3FAS) diet for 6 weeks before TB infection. Post-infection at 2 weeks, both groups were switched to an n-3 LCPUFA [eicosapentaenoic acid (EPA)/docosahexaenoic acid (DHA)] supplemented diet and euthanized at 4- and 14- days post-treatment. Iron and anemia status, bacterial loads, lung pathology, lung cytokines/chemokines, and lung lipid mediators were measured. Following 14 days of treatment, hemoglobin (Hb) was higher in the n-3FAD than the untreated n-3FAS group (*p* = 0.022), whereas the n-3FAS (drug) treated control and n-3FAS groups were not. Pro-inflammatory lung cytokines; interleukin-6 (IL-6) (*p* = 0.011), IL-1α (*p* = 0.039), MCP1 (*p* = 0.003), MIP1- α (*p* = 0.043), and RANTES (*p* = 0.034); were lower, and the anti-inflammatory cytokine IL-4 (*p* = 0.002) and growth factor GMCSF (*p* = 0.007) were higher in the n-3FAD compared with the n-3FAS mice after 14 days. These results suggest that n-3 LCPUFA therapy in TB-infected mice, in combination with TB medication, may improve anemia of infection more in low n-3 fatty acid status than sufficient status mice. Furthermore, the low n-3 fatty acid status TB mice supplemented with n-3 LCPUFA showed comparatively lower cytokine-mediated inflammation despite presenting with lower pro-resolving lipid mediators.

## Introduction

Tuberculosis (TB) disease is considered as an example of host immune failure (1), because of the ability of the pathogen to manipulate or evade the cellular immune responses to favor its persistence (2). TB is associated with a host inflammatory response, which results in injury to the surrounding tissue, causing significant chronic pulmonary impairment and morbidity (3–5). However, timely diagnosis and effective treatment of TB can limit infectivity and tissue damage due to the associated inflammatory effects that result in post-TB impairments, such as fibrosis and bronchiectasis, after curing (6, 7). Post-TB treatment lung impairment is a major TB-related disability burden (8). One of the common causes of this impairment is the inflammation associated with *Mycobacterium tuberculosis* (Mtb) infection (9). Thus, reducing inflammation may limit tissue damage and improve treatment, and other clinical outcomes (10). Hence, adjunctive TB therapies have been investigated frequently to augment and increase the success of standard TB treatment outcomes *via* immunomodulation (1, 11, 12). This host-directed immunotherapeutic treatment concept is designed to address the improvement of long-term outcomes and to promote a cure.

Over the past 20 years or more, the understanding of the functionality of dietary PUFAs not only as essential nutrients but their ability to also favorably modulate many disease parameters, particularly related to inflammation, has become more apparent (13). The n-3 long-chain polyunsaturated fatty acids (n-3 LCPUFAs) exert favorable health effects on several biological processes, including immune-modulation, making it a potential therapeutic agent toward combating inflammatory diseases (14–16). When supplemented in disease as a therapy, this compound has long been recognized to have anti-inflammatory activity, which has been observed in rheumatoid arthritis (17, 18), inflammatory bowel disease (19, 20), and respiratory conditions, such as asthma (21). The ability of n-3 LCPUFAs to down-regulate several mechanisms associated with inflammation, suggests that these FAs might be important in controlling the development and severity of inflammatory diseases, and they appear to be useful as components of novel therapy approaches (22), possibly also in patients with TB.

Consumption of n-3 PUFAs increases the phospholipid n-3 PUFA composition of immune cells and various tissues, thus, leading to the capacity to produce a pro-resolving lipid mediator (LM) profile upon a stimulus (23, 24). Immune cell n-3 PUFA composition is important as it influences immune functions, such as phagocytosis, neutrophil activity, and inflammatory responses (25, 26). LCPUFAs are incorporated even faster into leukocytes compared with erythrocytes, especially in an inflammatory or infectious milieu where there is rapid cell turnover (27, 28). Furthermore, pro-resolving LMs increased in plasma after 1 day of marine oil supplementation in healthy humans (29). These LMs are known as specialized pro-resolving mediators (SPMs) and include resolvins, protectins, and maresins, which contribute to inflammation resolution (14). The SPMs are known to reduce pro-inflammatory LMs, limit pro-inflammatory cytokine and chemokine production, and modify immune cell recruitment while stimulating the release of anti-inflammatory cytokines (14). An adequate presence of eicosapentaenoic acid (EPA) and docosahexaenoic acid (DHA) in cell membranes is also known to enhance phagocytosis of apoptotic cells and bacteria (30, 31), whereas the SPMs may assist in bacterial killing (14, 32). Increased EPA and DHA intake has also been shown to inhibit a wide range of inflammatory proteins, such as tumor necrosis factor (TNF), cyclooxygenase-2 (COX-2), and interleukin-6 (IL-6) (13, 33, 34).

Prior research has shown that a good n-3 PUFA status or supplementation prior to infection is not advantageous in TB infection (35–37). However, in a recent study from our group, we demonstrated that n-3 LCPUFA supplementation administered as therapy after TB infection lowered systemic and lung inflammation in *Mtb-*infected C3HeB/FeJ mice that did not receive TB drug treatment (38). Since some populations at risk for TB has low n-3 PUFA status (39, 40), we also previously investigated the effects of n-3 PUFA supplementation on low vs. sufficient n-3 PUFA mice without TB drug treatment (41). In this earlier study without TB medication, low n-3 PUFA status mice reacted differently and showed no benefit to supplementation after TB infection than mice with a sufficient n-3 PUFA status (35, 41).

Therefore, the current investigation focused to assess the effect of adjunct n-3 LCPUFA supplementation after infection on *Mtb-*infected C3HeB/FeJ mice with sufficient compared to low n-3 PUFA status, while being on standard TB drug treatment.

## Materials and Methods

### Animals and Ethics Statement

Male and female C3HeB/FeJ mice (Jackson Laboratory, Bar Harbour, ME), aged 10 to 12 weeks, were used for the experiment. Animals were housed at biohazard level 3 (BSL3) physical containment facilities at the Faculty of Health Sciences, University of Cape Town. After infection, mice were randomly placed into groups of six in standard type 2 long individually ventilated cages with filter tops, dried wood shavings, and shredded filter paper as floor coverings. Mice were housed under a 12/12 h light/dark cycle (lights on at 06:00) at 22 ± 2°C and 55 ± 10% relative humidity, with bodyweight measured weekly. The experiments were conducted according to the South African National Guidelines (SANS 10386:2008) and the University of Cape Town practice guidelines for laboratory animal procedures. The study protocol received approval from the AnimCare Animal Research Ethics Committee of North-West University, South Africa (Ethics number: NWU-00055-19-S5), and the Animal Research Ethics Committee of the University of Cape Town, South Africa (Ethics number: FHS AEC 019-023).

### Experimental Design

**Figure 1:**
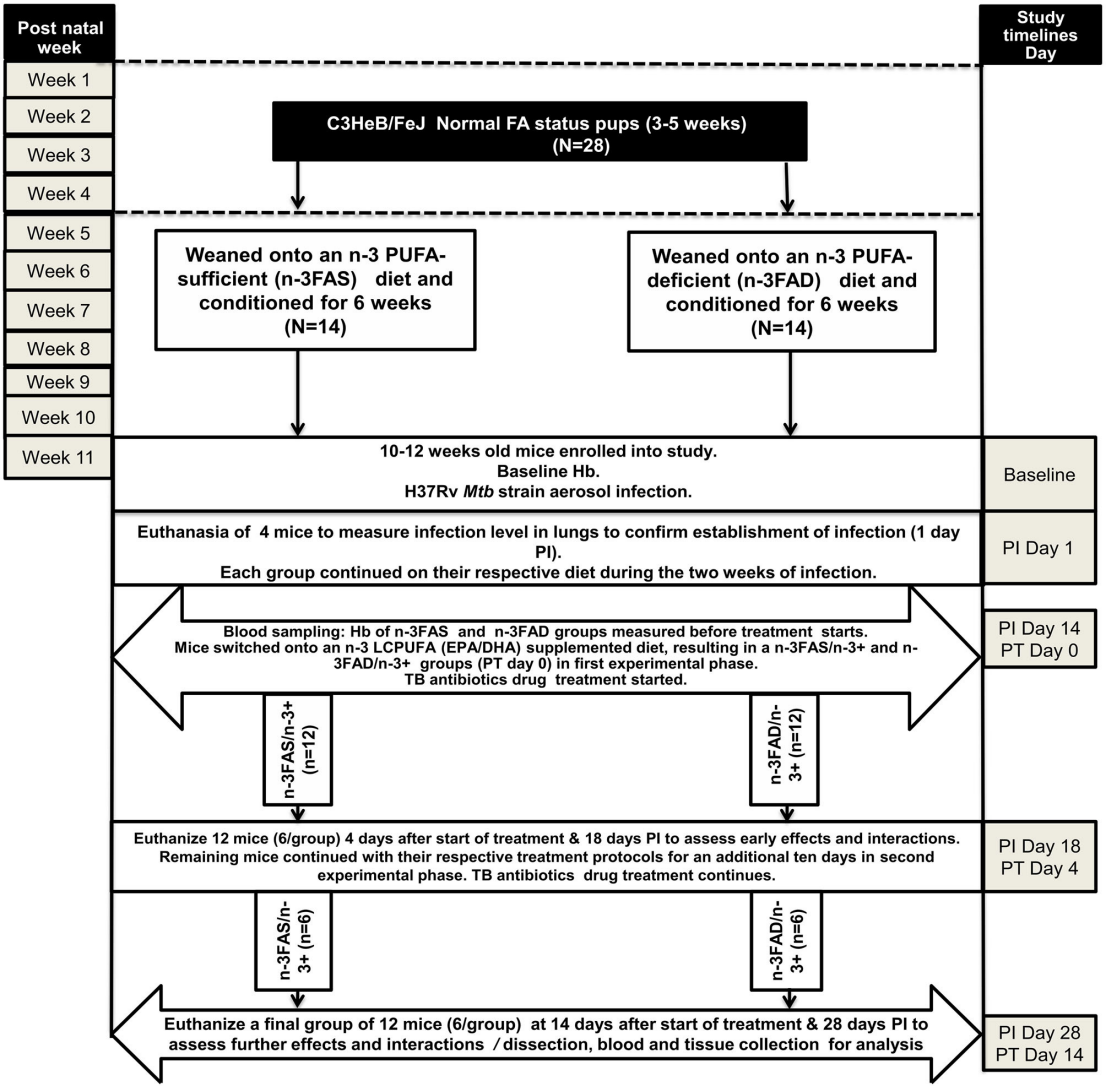
Experimental design of the study. EPA, eicosapentaenoic acid; DHA, docosahexaenoic acid; FA: fatty acid; N: number of mice; n-PUFA: omega-3 polyunsaturated fatty acid; n-LCPUFA: omega-3 long-chain polyunsaturated fatty acid; PT, posttreatment; PI, postinfection; n-3FAS, omega-3 fatty acid-sufficient; n-3FAD, omega-3 fatty acid-deficient; n-3FAS/n-3+: omega-3 fatty acid-sufficient on EPA/DHA supplemented diet; n-3FAD/n-3+: omega-3 fatty acid-deficient on EPA/DHA supplemented diet.

Figure 1 outlines the experimental design used for the study. Twenty-eight normal status 3–5 week-old pups were randomly weaned onto either an n-3 PUFA-deficient (n-3FAD) (*n* = 14) or n-3 PUFA-sufficient diet (n-3FAS) (*n* = 14) for 6 weeks, which was previously shown to produce mice with low and sufficient n-3 PUFA red blood cell (RBC) status, respectively (35, 41). Prior to infection, baseline hemoglobin (Hb) was measured. One day postinfection, four mice were euthanized to confirm the infection dose. Each group continued on their respective diets during the 2 weeks of infection, and thereafter, they switched onto an n-3 LCPUFA (EPA/DHA) supplemented diet, corresponding to the n-3FAS/n-3+ (*n* = 12) and n-3FAD/n-3+ (*n* = 12) experimental groups. Both groups also started TB antibiotics treatment at that same time. Prior to the diet switch onto the n-3 supplemented diet, pretreatment Hb was also measured to determine post infection Hb status. A total of 12 mice, 6 from the n-3FAS/n-3+ group and 6 from the n-3FAD/n-3+ group, were euthanized 4 days posttreatment in the first experimental phase (Phase 1). The remaining 12 mice, 6 of each group continued with their respective treatment protocols for an additional 10 days until euthanasia at day 14 after the initial treatment commenced, representing Phase 2 of the experimental design, as described in Figure 1. The body weight of the mice was measured weekly and mice had *ad libitum* access to supplemented diets and water. All mice were on standard TB antibiotics Rifafour® for 4 days of treatment (Phase 1), then rifampicin and isoniazid (RH) for the remaining 10 days (Phase 2). All anti-TB drugs used for treatment were administered either by esophageal gavage or in the drinking water. The following doses were used: in Phase 1 (intensive phase), each mouse received 0.2 ml of antibiotic consisting of Rifafour^®^ (150mg rifampicin + 75 mg isoniazid + 400 mg pyrazinamide + 275 mg ethambutol) dissolved in 30 ml distilled water through oral gavage administration daily. In Phase 2 (continuation phase), isoniazid (0.1 g/L) and rifampicin (0.1 g/L) were administered to mice *via* their drinking water. The water consumption was measured in Phase 2 to confirm equal drug intake in all two groups.

### Experimental Diet Composition of Mice

All diets were isocaloric and contained 10% fat, with modifications of the fat source depending on whether it was a sufficient, deficient, or supplementation diet. All diets were a purified American Institute of Nutrition (AIN)-93G (42) laboratory diet formulation (commercially obtained from Dyets Inc. Bethlehem, USA). The basal AIN-93G-formulated FAS diet contained soybean oil at 70 g/kg diet and hydrogenated coconut oil at 30 g/kg diet (43). The FAD diet contained hydrogenated coconut oil at 81 g/kg diet and safflower oil at 19 g/kg diet (44–46). The n-3 LCPUFA supplementation diet fat composition contained soybean oil at 70 g/kg diet, coconut oil at 27 g/kg diet, and commercially obtained Incromega TG4030 oil (Croda Chemicals, Snaith, Europe), DHA 500 TG SR with a minimum of 44% FAs as EPA and a minimum of 28% FAs as DHA at 3 g/kg diet (38), as described in Supplementary Table 1. All diets were custom prepared in pellet form and stored at −20°C until use. Diets were thawed in batches, by placing them into a refrigerator (4°C). Once the bags had been opened, the diets were placed in an airtight container. The pellets were weighed weekly to determine the actual amount of food consumed. The diet was administered according to the description in Figure 1 above.

### Aerosol Infection

Virulent *Mtb* H37Rv strain was cultured and stocks were prepared and stored at −80°C, as described previously (38, 47). Mice were infected *via* aerosol by nebulizing with 6 ml of a suspension that contained 2.4 × 10^7^ live bacteria in an inhalation exposure system (model A4224, Glas-Col) for 40 min. The mice were each infected with approximately 50–70 *Mtb* colony-forming units (CFU).

### Blood and Tissue Collection

At the end of each phase of treatment, mice were euthanized by exposure to halothane, after which blood was collected *via* cardiac puncture. The blood was collected into ethylenediaminetetraacetic acid (EDTA)-coated Microtainer^®^ tubes (K_2_EDTA, 1000 μl, BD), Hb was measured in whole blood and then centrifuged at 8,000 rpm for plasma collection. The plasma was used for cytokine analyses and peripheral blood mononuclear cells (PBMCs) were collected for FA analysis. The liver, spleen, and lung lobes were removed aseptically and weighed before preparation. The left lung lobe and spleen were homogenized in saline and 0.04% Tween-80 for the analysis of the bacillary load and lung cytokines. The right superior and postcaval lung lobes and the liver were snap-frozen in liquid nitrogen and stored at −80°C for lung LM and liver iron analyses, respectively. The right middle lobe was fixed in 10% neutral buffered formalin for immunohistochemistry analysis. As a rough measure of inflammation, the lung- and spleen-weight-indexes were determined at the end of each treatment phase by taking the square root of ratios of spleen or lung tissue weight to endpoint (euthanasia) bodyweight of mice and multiplying it by 10 (47).

### Hemoglobin and Liver Iron Analysis

Hemoglobin concentrations were measured in tail vein whole blood in live mice and after euthanasia blood collection in whole blood using a portable HemoCue^®^ Hb 201+ photometer (HemoCue AB, Angelholm, Sweden). Liver iron was analyzed at the Central Analytical Facilities, Stellenbosch University, SA, on an Agilent 7900 quadrupole ICP-MS in He collision mode. The National Institute of Standards and Technology (NIST) traceable standards were used for calibration, and the accuracy of the method was verified using the certified reference material Seronorm L2, prepared in the same way as the samples. Two n-3 FA sufficient control groups, untreated (*n* = 6) and treated with TB drugs (*n* = 6) were included in the Hb and liver iron analysis. Results for n-3 FA sufficient uninfected mice (*n* = 6) are also presented.

### Immune Cell Total Phospholipid Fatty Acid Composition Analysis

The total phospholipid FA composition of peripheral blood mononuclear cell (PBMC) was analyzed by gas chromatography-tandem mass spectrometry as previously described (38). FAs were extracted from ~200 μl PBMCs with chloroform: methanol (2:1, v:v; containing 0.01% butylated hydroxytoluene (BHT)) by a modification of the method by Folch et al. (48). Phospholipids were separated by thin-layer chromatography (TLC) (Silica gel 60 plates, Merck). The composition of EPA (20:5n-3), DHA (22:6n-3), arachidonic acid (AA, 20:4n-6), osbond acid (22:5n-6), total n-3 LCPUFA, total n-6 LCPUFA, and total n-6/n-3 LCPUFA ratio were then determined as a percentage of total phospholipid FAs. This was computed by taking the concentration of a particular FA as a percentage of the total concentration of all FAs identified in the sample.

### Bacterial Load and Histopathology

The bacterial loads of the lung and spleen were determined at euthanasia, at the end of each treatment phase (4- and 14- days posttreatment). The left lobes of the lung and spleens of each mouse were removed aseptically, weighed, homogenized, and plated onto Difco™ Middlebrooks 7H10 Agar (BD Biosciences) medium with 10% oleic acid-albumin-dextrose-catalase (OADC) supplementation. The CFU was determined at 21 days following incubation at 37°C. The data are expressed as log_10_ CFU. For lung histopathology analysis, the right middle lobes of the lungs were dissected and fixed in 4% neutral buffered formalin. The fixed tissue was processed using the Leica TP 1020 Processor for 24 h and then embedded in paraffin wax. Leica sliding microtome 2000R was used to cut 2 μm thick sections of the embedded tissues. Three sections with 30 μm distance apart per tissue were obtained, deparaffinized, and stained with hematoxylin/eosin (H&E) stain. Images of the H&E slides were acquired in Nikon Eclipse 90i microscopes and analyzed with NIS-Elements AR software (Nikon Corporation, Tokyo, Japan) to ascertain the granulomatous area and free alveolar air space as a percentage of the total lung tissue as previously described (49).

### Lipid Mediator Analysis

Lipid mediators in crude lung homogenates were extracted and analyzed by liquid chromatography-tandem mass spectrometry. LMs were extracted from lung tissue, in 10 μl/mg homogenization buffer (phosphate-buffered saline), with solid-phase extraction (SPE) using Strata-X (Phenomenex, Torrance, CA). The method was modified for Strata-X SPE columns from a previously described method (50). Data were quantified with Masshunter B0502, using external calibration for each compound and internal standard [PGD2-d4, PGE2-d4, PGF2-d4, and 5- and 12-HETE-d8; 1,000 pg of each (Cayman Chemicals, Ann Arbor, MI)] to correct for losses and matrix effects. Extracted and quantified LMs included: DHA-derived pro-resolving 17-hydroxydocosahexaenoic acid (17-HDHA) and protectin D1 (PD1); EPA-derived pro-resolving prostaglandin E3 (PGE3) and LM intermediates 2-, 5-, 9-, 11-, 15-, and 18-hydroxyeicosapentaenoic acid (HEPE); AA-derived pro-inflammatory intermediates 5-, 8-, 9-, 11-, 12-, and 15-hydroxyeicosatetraenoic acid (HETE); AA-derived prostaglandin D2 (PGD2), prostaglandin E2 (PGE2), and prostaglandin F2α (PGFα2); and thromboxane B2 (TBXB2).

### Cytokine Analysis

Lung cell-free homogenates were prepared using the left lung lobe by homogenizing with 0.04% Tween-80 in saline. The crude homogenate was centrifuged at 3,000 *g* for 5 min, filtered, and then the supernatant was stored at −80°C until analysis. After thawing, samples were centrifuged at 6,000 rpm in a microcentrifuge for 30 min at 4°C, before mouse enzyme-linked immunosorbent assay kits were used to measure cytokines. Quansys Biosciences Q-Plex™ Mouse Cytokine Screen (West Logan, WV) Q-Plex Array 16 plex was then used to measure cytokines, which included IL-1α, IL-1β, IL-2, IL-3, IL-4, IL-5, IL-6, IL-10, IL-12, IL-17, monocyte chemoattractant protein 1 (MCP-1), interferon-gamma (IFN-γ), tumor necrosis factor-alpha (TNF-α), granulocyte-macrophage colony-stimulating factor (GM-CSF), macrophage inflammatory protein 1-alpha (MIP-1α), and regulated on activation normal T-cell expressed and secreted (RANTES) in the supernatant. Arrays were analyzed using the Q-View Imager Pro and Q-View Software (Quansys Biosciences Q-Plex^™^, West Logan, WV).

### Statistical Analysis and Graphical Representation

All statistical analyses and graphics were performed with GraphPad Prism Software version 8.2 (GraphPad Software Inc., La Jolla, CA, USA). A two-sided alpha of 0.05 and a power of 80% was used to estimate a minimum sample of *n* = 6 as previously described (51). The normality of the data was evaluated by histogram visual inspection and the Kolmogorov-Smirnov test. Results are presented as means ± SE of the mean. Differences for Hb and liver iron among groups were analyzed with ANCOVA and *post-hoc* Tukey tests. Differences between the same treatments (euthanized groups) on days 4 and 14 were analyzed with independent two-tailed *t*-tests. Differences for all other markers between n-3FAD and n-3FAS groups were analyzed using independent two-tailed *t*-tests. Statistically significant differences are presented as follows: ^*^p < 0.05; ^**^p < 0.01; ^***^p < 0.001.

## Results

### Diet Intake and Bodyweight

There were no differences between groups in the daily food intakes throughout the experiment (n-3FAS/n-3+, 3.51 ± 0.14 g; n-3FAD/n-3+, 3.74 ± 0.25). As expected, there was no significant difference in weight gain between the groups after 4 and 14 days of n-3 LCPUFA supplementation (data not shown), indicating that differential food intake between the groups may not be an underlying reason for the observed results.

### Indices of Iron Status and Anemia

**Figure 2:**
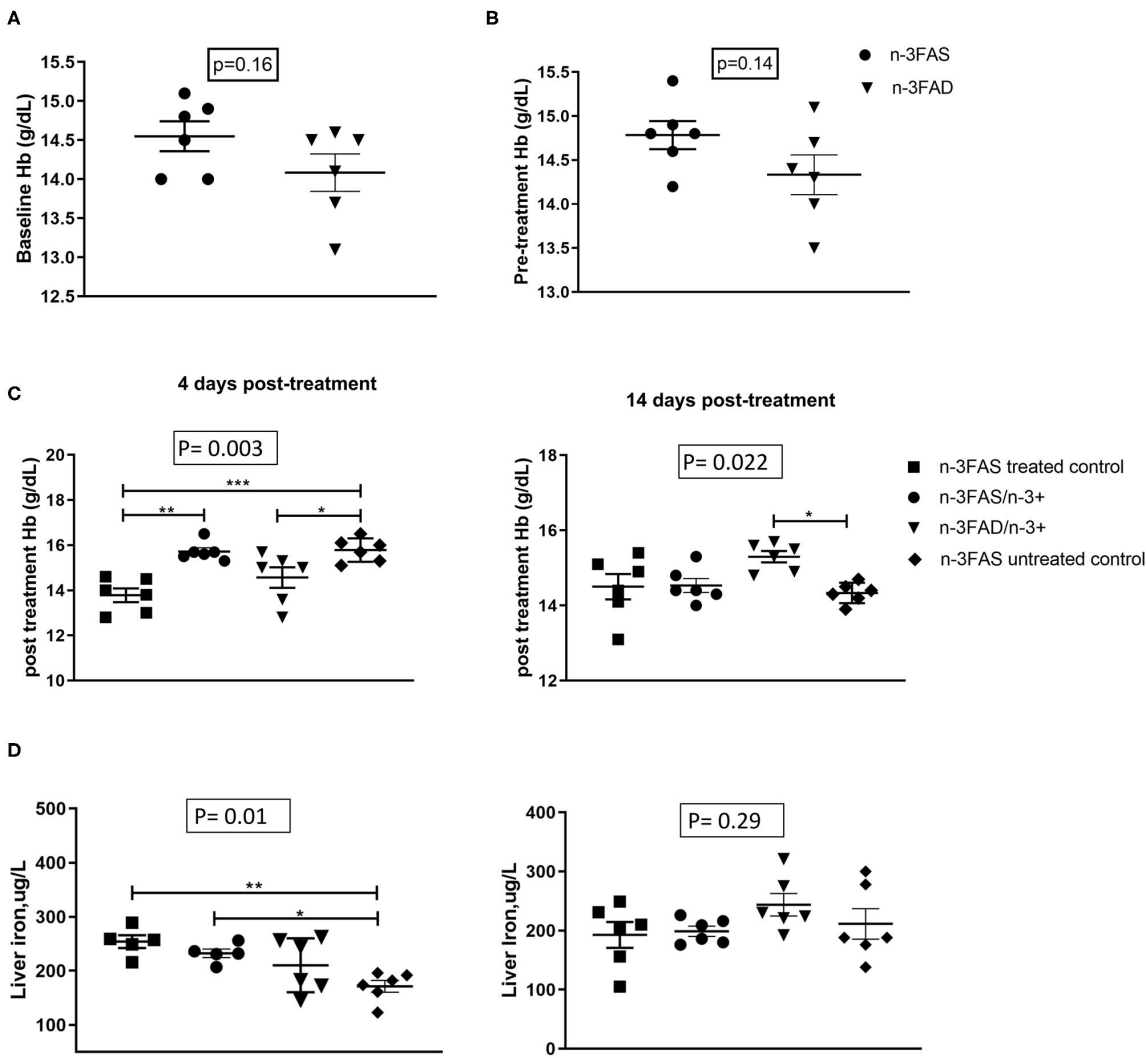
Effect of status and n-3 long-chain polyunsaturated fatty acids (LCPUFA) supplementation on indices of anemia, **(A)** Baseline hemoglobin (Hb) levels, **(B)** Pretreatment Hb levels, **(C)** Posttreatment Hb levels, and **(D)** liver iron levels in liver homogenates. Baseline Hb levels were measured after 6 weeks of conditioning to either sufficient or low n-3 PUFA status. Pretreatment Hb levels measured after TB infection but prior to commencement of EPA/DHA supplementation. Posttreatment Hb levels were measured at each euthanasia time point after EPA/DHA supplementation. The data are represented as mean ± SEM of *n* = 6 mice/group and representative of two independent experiments. Independent *t*-tests were used to compare means, significance at ^*^p < 0.05, ^**^p < 0.01, ^***^p < 0.001; n-3FAS, omega-3 fatty acid- sufficient diet group; n-3FAD, omega-3 fatty acid-deficient diet group; /, switched to; n-3FAS, omega-3 fatty acid-sufficient; n-3FAS/n-3+, omega-3 fatty acid-sufficient group switched to DHA/EPA-enriched diet; n-3FAD/n-3+, omega-3 fatty acid-deficient group switched to DHA/EPA-enriched diet; n-3FAS treated control, omega-3 fatty acid-sufficient group on only tuberculosis (TB) antibiotics treatment; n-3FAS untreated control, omega-3 fatty acid-sufficient group infected with TB but not on either TB antibiotics treatment or DHA/EPA-enriched diet supplementation.

There were no significant differences in the baseline (before infection) and pretreatment (before EPA/DHA supplementation) Hb levels between the n-3FAD and the n-3FAS groups (Figures 2A,B). Figure 2C shows that, between days 4 and 14 after treatment, Hb decreased in untreated n-3FAS control mice from 15.8 ± 0.5 to 14.3 ± 0.3 (*d* = 0.4, *p* < 0.001), decreased in n-3FAS mice from 15.7 ± 0.4 to 14.5 ± 0.5 (*d* = 0.4, *p* = 0.001) but tended to increase in n-3FAD mice from 13.8 ± 1.4 to 14.9 ± 0.6 (*d* = 1.1, *p* = 0.07), and had a nonsignificant increase in treated n-3FAS controls from 13.8 ± 0.8 to 14.4 ± 0.8 (*d* = 0.8, *p* = 0.148). Uninfected control mice (*n* = 6) had a Hb of 15.0 ± 0.5 (results not shown). Following 14 days of treatment, Hb was higher in the n-3FAD than the n-3FAS untreated controls (*p* = 0.022, Figure 2C), whereas the n-3FAS (drug) treated controls and n-3FAS group were not. These results suggest that TB-induced anemia is mitigated more by n-3 LCPUFA supplementation in the low n-3 PUFA status mice than in the sufficient status groups with and without additional n-3 LCPUFA supplementation. No significant differences were seen in the liver iron concentrations between the groups after 14 days of treatment (Figure 2D).

### Lung Cytokines/Chemokines

**Figure 3:**
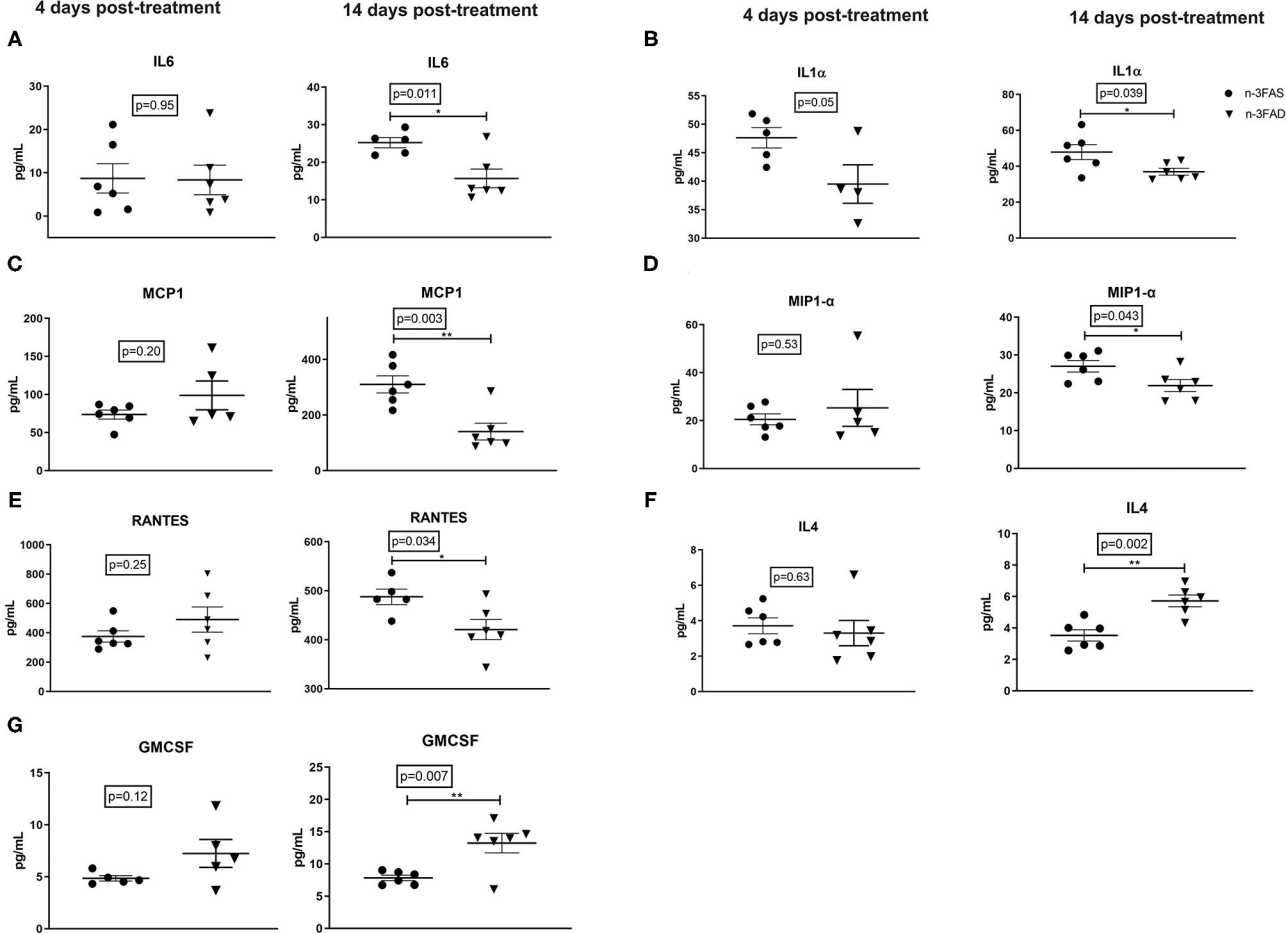
Effects of conditioning and treatment on lung cytokine levels. **(A)** IL-6, **(B)** IL-1α, **(C)** MCP-1, **(D)** MIP-1α, **(E)** RANTES, **(F)** IL-4, and **(G)** GM-CSF. The data are represented as mean ± SEM of n = 6 mice/group and representative of two independent experiments. Unpaired two-tailed *t*-tests were used to compare means, significance at ^*^p < 0.05, ^**^p < 0.01. IL, interleukin; IL-1α, interleukin 1 alpha; MIP-1 α, macrophage inflammatory protein 1-alpha; MCP-1, Monocyte chemoattractant protein-1; GM-CSF, granulocyte-macrophage colony-stimulating factor; RANTES, Regulated on Activation, Normal T Cell Expressed and Secreted; n-3FAS, omega-3 fatty acid- sufficient diet group; n-3FAD, omega-3 fatty acid-deficient diet group.

Lung IL-6 (*p* = 0.011, Figure 3A), IL-1α (*p* = 0.039, Figure 3B), MCP1 (*p* = 0.003, Figure 3C), MIP1- α (*p* = 0.043, Figure 3D), and RANTES (*p* = 0.034, Figure 3E) were lower in the n-3FAD group when compared to the n-3FAS group, respectively, after 14 days of treatment with n-3 LCPUFA supplementation. These results suggest that a low n-3 FA status supplemented with n-3 LCPUFA as an adjunct anti-TB therapy has comparatively better inflammation-lowering effects compared with mice with a prior n-3 FA sufficient status. The reduction in pro-inflammatory cytokines showed a concomitant increase in anti-inflammatory cytokine IL-4 (*p* = 0.002, Figure 3F) and hematopoietic growth factor GM-CSF (*p* = 0.007, Figure 3G) in the n-3FAD group compared to the n-3FAS group after 14 days of treatment. This result suggests that the adjunct n-3 LCPUFA supplementation in the n-3FAD group showed a better lung anti-inflammatory response than in the n-3FAS group.

### Tuberculosis-Associated Outcomes

**Figure 4:**
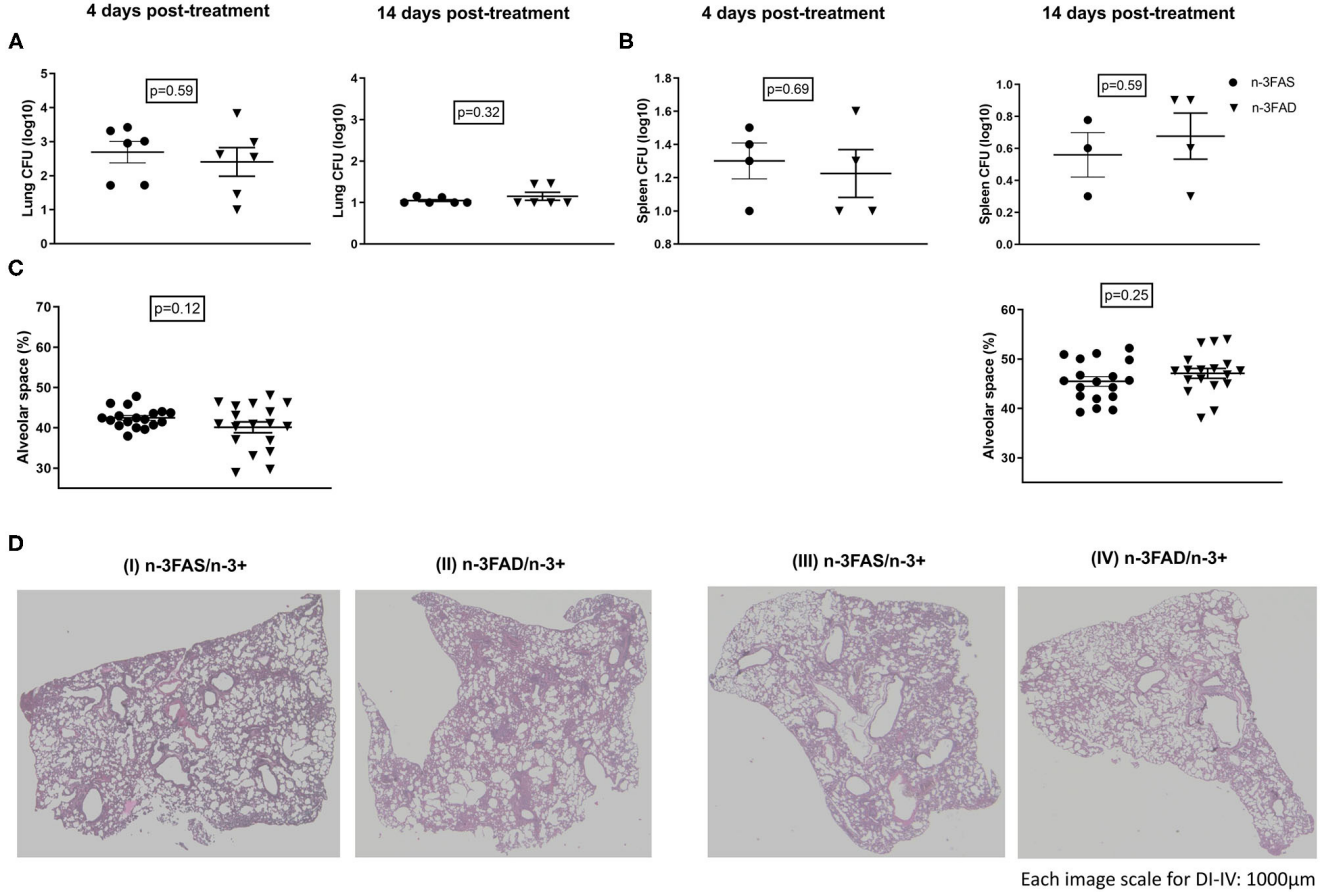
Effect of treatments on lung bacillary load and lung histopathology according to fatty acid status. **(A)** Lung bacteria load, **(B)** spleen bacteria load, **(C)** percentage free alveolar space, and **(D)** hematoxylin–eosin-stained sections of the lungs of selected representative group, after 4 [**(D)** (I,II)] and 14 [**(D)** (III,IV)] days treatment period. The data represented as mean ± SEM of *n* = 6 mice/group and representative of two independent experiments. Unpaired two-tailed *t*-test was used to compare means, significance at ^*^p < 0.05, ^**^p < 0.01, ^***^p < 0.001; n-3FAS, omega-3 fatty acid-sufficient diet group; n-3FAD, omega-3 fatty acid-deficient diet group.

We investigated bacterial loads, free alveolar space, and H&E stained histology sections of *Mtb-*infected mice, after 2 weeks of the dietary intervention. There were no significant differences in the lung and spleen bacteria loads between the n-3FAD and the n-3FAS supplemented groups (Figures 4A,B). There was a trend of less free alveolar space in the n-3FAD than the n-3FAS groups comparatively at the early stage of treatment (Phase 1) [4 days posttreatment; 42 ± 1% vs. 40 ± 1%, Figures 4C,D (I,II)], however, the n-3FAD group measured more free alveolar space at the latter stage of treatment (Phase 2) than the compared n-3FAS group [14 days posttreatment; 47 ± 1% vs. 45 ± 1%, Figures 4C,D (III,IV)] although not significantly so. This indicates that in the presence of anti-TB drugs, no difference in bacterial clearance in the FAD and FAS groups due to n-3 FA supplementation is evident.

### Lung Lipid Mediators

**Figure 5:**
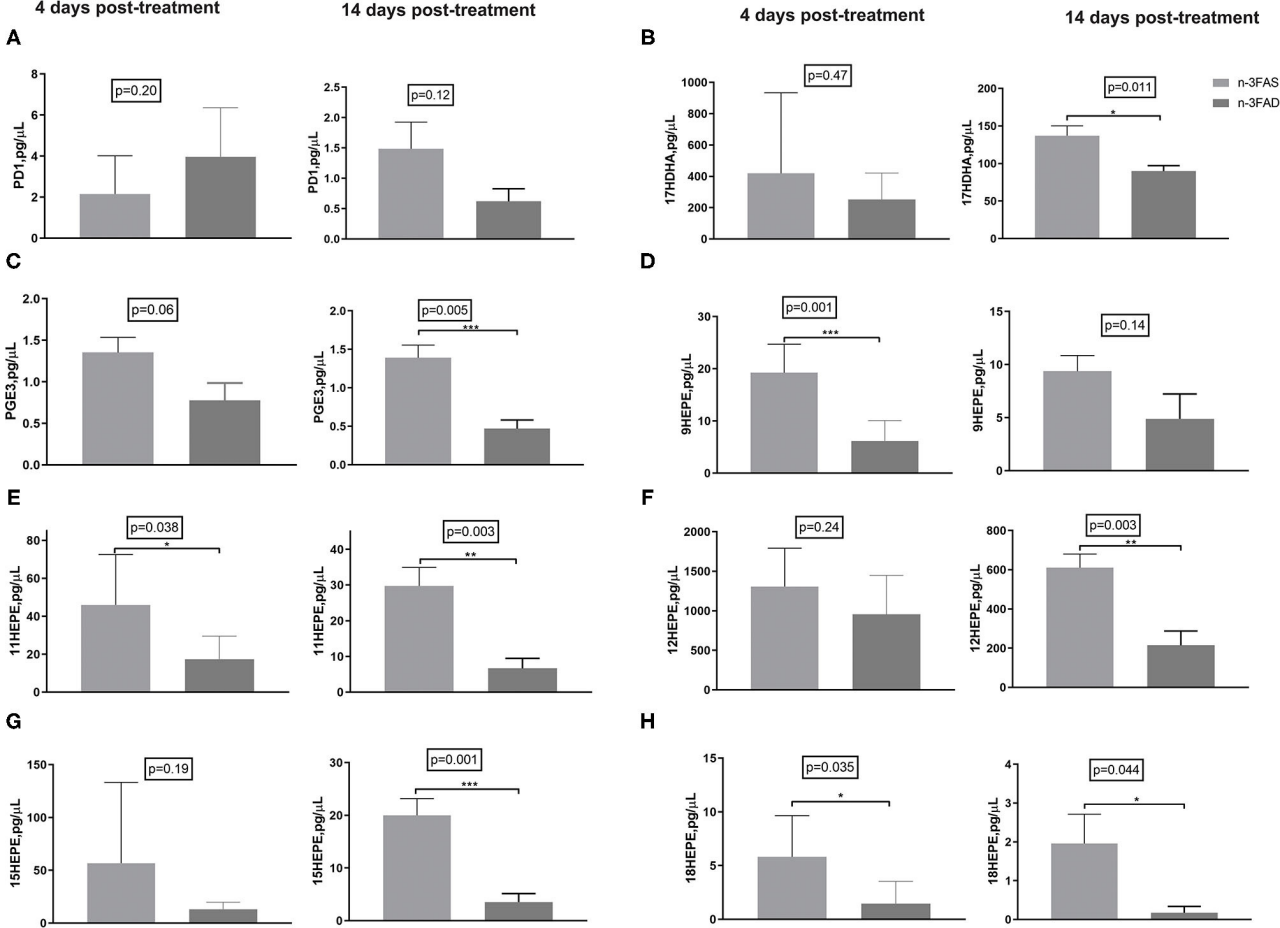
Effects of conditioning on lipid mediators in crude lung homogenate at the local site of intervention **(A)** PD1, **(B)** 17-HDHA, **(C)** PGE3, **(D)** 9-HEPE, **(E)** 11-HEPE, **(F)** 12-HEPE, **(G)** 15-HEPE, and **(H)** 18-HEPE. The data are represented as mean ± SEM of *n* = 6 mice/group and representative of two independent experiments. Unpaired two-tailed *t*-tests were used to compare means, significance at ^*^p < 0.05, ^**^p < 0.01, ^***^p < 0.001. PGE3, Prostaglandin E3; PD1, Protectin D1; HDHA, hydroxydocosahexaenoic acid; HEPE, hydroxyeicosapentaenoic acid; n-3FAS, omega-3 fatty acid-sufficient diet group; n-3FAD, omega-3 fatty acid-deficient diet group.

Docosahexaenoic acid -derived pro-resolving PD1 showed no significant comparative differences between the groups, neither at 4 days nor at 14 days posttreatment; however, a trend of progressively reduced levels was seen in the 14 days posttreatment samples (*p* = 0.12; Figure 5A). The n-3FAS group showed comparatively higher concentrations of pro-resolving 17HDHA and PGE3 than the n-3FAD group after 14 days of treatment (*p* = 0.011, and *p* = 0.005, respectively; Figures 5B,C). Similarly, higher concentrations of EPA-derived pro-resolving intermediates; 9-HEPE (4 days PT, *p* = 0.001, Figure 5D), 11-HEPE (14 days PT, *p* = 0.003, Figure 5E), 12-HEPE (14 days PT, *p* = 0.003, Figure 5F), 15-HEPE (14 days PT, *p* = 0.001, Figure 5G), and 18- HEPE (14 days PT, *p* = 0.044, Figure 5H), were observed in the n-3FAS group when compared to the n-3FAD group. The pro-inflammatory AA-derived PGF2 and PGD2 were significantly higher in the n-3FAD group after 14 days of treatment (*p* = 0.001 and *p* = 0.004, respectively, Supplementary Figures 1A,B), comparatively. No significant differences were seen in the concentrations of AA-derived pro-inflammatory intermediates 5-, 8-, 9-, 11-, 12-, and 15-HETE (Supplementary Figures 1C–H) among the n-3FAS and the n-3FAD groups.

### Effects of Treatments on PBMC Fatty Acid Composition

The phospholipid FA compositions of PBMCs after 4 and 14 days of n-3 LCPUFA supplementation are presented in Table 1. The n-3FAS group had comparatively higher EPA, DHA, and n-3 LCPUFA compositions than the compared n-3FAD supplemented groups (4 days PT, all *p* < 0.001 and 14 days PT, all *p* < 0.001). In addition, the composition of AA, osbond acid, total n-6 LCPUFAs, and the total n-6/n-3LCPUFA ratio were higher in the n-3FAD groups in comparison to the n-3FAS groups after 4 days (*p* < 0.001) and 14 days (*p* < 0.001) of n-3 LCPUFA supplementation.

**Table 1:** Effects on PBMC phospholipid fatty acid composition in sufficient and low n-3 PUFA status C3HeB/FeJ mice infected with *Mtb* after receiving n-3 LCPUFA supplementation^#^ (*n* = 48).

**4 days posttreatment** ^*^				**14 days posttreatment** ^**^		
**Fatty acids**	**n-3FAS**	**n-3FAD**	***P*-value**	**n-3FAS**	**n-3FAD**	***P*-value**
20:5n-3 (EPA)	0.60 ± 0.02	0.30 ± 0.02	<0.001	0.74 ± 0.08	0.16 ± 0.04	<0.001
22:6n-3 (DHA)	11.54 ± 0.19	10.25 ± 0.40	<0.001	12.19 ± 0.37	9.22 ± 0.34	<0.001
Total n-3 LCPUFA	12.99 ± 0.17	10.93 ± 0.39	<0.001	14.12 ± 0.38	9.97 ± 0.38	<0.001
20:4n-6 (AA)	18.38 ± 0.38	21.08 ± 0.39	<0.001	17.24 ± 0.24	21.52 ± 0.25	< 0.001
22:5n-6 (Osbond)	0.89 ± 0.04	2.54 ± 0.20	<0.0001	1.07 ± 0.03	3.38 ± 0.26	<0.0001
Total n-6 LCPUFA	23.22 ± 0.46	27.54 ± 0.79	<0.001	22.51 ± 0.22	29.71 ± 0.46	<0.001
Total n-6/n-3LCPUFA ratio	1.79 ± 0.03	2.55 ± 0.15	<0.001	1.60 ± 0.05	3.01 ± 0.17	<0.001

# *Data are reported as means ± SEM percentage of total fatty acids of n = 6 mice/group and representative of two independent experiments. Unpaired two-tailed t-tests were used to test the effects between groups. AA, Arachidonic acid; DHA, docosahexaenoic acid; EPA, eicosapentaenoic acid; LCPUFA, long-chain polyunsaturated fatty acids; n, the total number of mice used in the experiment; PBMC, peripheral blood mononuclear cell*;

* *, all groups were on standard TB antibiotics (Rifafour^®^)*;

** *, all groups were on standard TB antibiotics rifampicin and isoniazid (RH); n-3FAS, omega-3 fatty acid-sufficient diet group; n-3FAD, omega-3 fatty acid-deficient diet group*.

## Discussion and Conclusion

Our results indicate that adjunct n-3 LCPUFA supplementation in drug-treated Mtb-infected mice mitigate anemia of infection more in low n-3 FA status than sufficient status mice. This is remarkable as it was previously shown that, when not treated with TB drugs, supplementing low status n-3 FA mice with n-3 LCPUFA post-TB infection showed no benefit (35, 41). Protection against anemia during TB without making iron available for the pathogen is of utmost clinical value. Furthermore, as populations at risk for TB may have a low n-3 FA status, these results are promising to support n-3 LCPUFA as an adjunct therapy to TB drugs post-TB infection particularly in these populations. These results are in parallel with the lower pro-inflammatory cytokine/chemokine responses, particularly IL-6, and higher anti-inflammatory IL-4 levels in the mice with previously low n-3 FA status. The aforementioned observation occurred although the low n-3 FA status mice (n-3FAD group) had comparatively less EPA, DHA, and n-3 LCPUFA compositions, and concurrent comparatively lower concentrations of EPA- and DHA-derived LMs than the sufficient status mice (n-3FAS group), after supplementation.

A study done in Brazil on patients with TB suggests that anemia could be a biomarker of TB severity and that anemia was more frequent in the most severe clinical forms of this disease, such as meningeal and disseminated TB (52). The mitigation of iron deficiency and anemia in our study is likely due to the comparatively better inflammation-lowering effect (indicated by the reduced pro-inflammatory cytokines) in the FAD group. Anemia of infection is due to a cytokine-mediated defense against microbial pathogens, which acts by effectively withholding iron from microbes, thus depriving erythroid precursors of their iron supply (53, 54). It has been demonstrated that reducing IL-6, reduces hepcidin and stops iron sequestering due to infection (55), thus mitigating iron deficiency and anemia. Omega-3 PUFA has been shown to influence iron metabolism *via* improved membrane fluidity, subsequently increasing iron uptake, improving intracellular activity (56), and improving iron stores (57).

An inflammation lowering effect in lungs of patients with TB is beneficial and a highly desirable objective during treatment, since it would improve the chances for a more favorable long-term outcome and lung health in these patients. In this study, the reduced levels of lung IL-6, IL-1α, MCP1, MIP1-α, and RANTES, seen in the FAD group when compared with the FAS group, suggest comparatively less inflammation in the initially low n-3 FA status group, when supplemented with n-3 LCPUFA. The inflammation-lowering effect observed was supported by the significantly higher concentrations of anti-inflammatory cytokine IL-4 seen in the FAD group after 14 days of treatment. The elevated levels of GM-CSF in the same FAD group is also an interesting observation since GM-CSF can increase the proliferation and phagocytic capacity of alveolar macrophages (58), necessary in granuloma formation and containment of mycobacteria (59). GM-CSF can also contribute to mycobacterial containment by polarizing macrophages into a more Mtb-restrictive phenotype (60). Combined, these findings suggest Mtb-infected mice with a low FA status, supplemented with n-3 LCPUFAs, respond better than mice with initially sufficient status. This finding is important, especially if the target intervention population is known to be at high risk of low n-3 PUFA status. In addition, considering the major economic burden associated with pulmonary TB, the use of low-cost adjunctive therapies, such as omega-3 for such intervention, could be regarded as a cost-effective manner for improving treatment outcomes.

Despite the n-3FAS group showed comparatively a better pro-resolving LM profile (supported by their higher n-3 PUFA PBMC phospholipid composition), no reduction of its pro-inflammatory cytokine concentrations as compared to the FAD group was observed. Similar to our findings, the administration of fish oil has been shown to alter pro-resolving LMs, without significantly changing the cytokine concentrations in bronchoalveolar lavage fluid of rats (61). This could be explained by the effect of n-3 LCPUFAs supplementation on Th1/Th2 balance, and the inhibition of the Th2 type cytokine, IL 4 (62). Considering that resolving the inflammatory response in TB is a key in limiting tissue damage, an increase in pro-resolving mediators was expected to lead to resolution of inflammation and subsequent improvement in lung pathology (63, 64), also not apparent from our data. However, even though the FAD group presented with lower concentrations of the more biologically active EPA and DHA, which have been associated with improved health outcomes (16), adaptations in the low-status state may have led to a better inflammatory response in this group when supplemented. A possible explanation may be that mice with the deficiency status may be better primed for improved n-3 LCPUFA utilization due to their prior deficiency status (65).

We previously showed that EPA/DHA supplementation in mice with sufficient n-3 PUFA status did not interfere with the TB drug treatment when coadministered as an adjunct therapy (66). A similar trend was observed when mice with low n-3 PUFA status were supplemented with EPA/DHA, suggesting that the coadministration of EPA/DHA together with the currently used TB antibiotics has no observable adverse effects. Multidrug combination therapy approaches for TB treatment make it a candidate for possible drug-drug interactions (DDIs) or drug-nutrient interactions (DNIs) (67), which subsequently may result in poor TB treatment outcomes and the emergence of drug-resistant TB (68, 69). Considering the recent interest and the prospects associated with the use of repurposed drugs and pharmaconutrients as an adjunct therapy in TB (35, 70–72), this study further reinforces the prospect for the use of n-3 LCPUFA for such purposes, as was demonstrated recently by our group to lower systemic and lung inflammation in *Mtb-*infected mice (38). This result was contrary to the findings of Bonilla et al. however, who indicated that the endogenous production of n-3 PUFAs in fat-1 mice increases their susceptibility to TB. The researcher argued that the n-3 PUFAs impaired macrophage activation and diminished the antimycobacterial response in these cells from fat-1 mice (37), hence suggesting that n-3 PUFA–supplemented diets might have a detrimental effect on immunity to *Mtb*, which raises concerns about the safety of n-3 dietary supplementation in humans with TB. However, it should be noted that transgenic fat-1 mice can produce extensive amounts of n-3 PUFAs and drastically imbalance n-6/n-3 ratios. Furthermore, the timing of the supplementation is essential and should be done after infection, which was not possible with Bonilla's experiment. Based on our results, we indicate that supplementary intake of n-3 PUFAs is perhaps physiologically a better approach than using anti-inflammatory drugs to improve TB treatment outcomes.

The strengths of this study include the following: (1) this investigation took into consideration the different time points, and hence the different phases of the inflammatory and immune response; (2) this study used the same combinations of standard TB treatment is currently used in humans; (3) this study also used a murine model that reflects human pulmonary TB pathology. A possible limitation of our study was that there were no control groups that were not supplemented with adjunct n-3 LCPUFA; however, our goal in this instance was to investigate whether supplementation response in TB was dependent on n-3 PUFA status only. Considering that populations at risk for TB may have either a low or sufficient n-3 PUFA status, it was prudent to investigate the effects of both these n-3 PUFA statuses, in combination with TB medication, before proceeding to human trials.

In conclusion, when EPA/DHA was administered post-TB infection as a treatment adjunct to standard TB medication, anemia of infection was mitigated more in the low n-3 PUFA status mice than in the sufficient status mice. It also resulted in a lower production of pro-inflammatory cytokines, while increasing the anti-inflammatory cytokines in mice with low n-3 FA status. Thus, adjunct n-3 LCPUFA therapy for TB disease shows promise for improving anemia of infection and inflammation-related clinical outcomes, particularly in those with low n-3 PUFA status.

## Data Availability Statement

The raw data supporting the conclusions of this article will be made available by the authors, without undue reservation.

## Ethics Statement

The experiments were conducted according to the South African National Guidelines (SANS 10386:2008) and the University of Cape Town practice guidelines for laboratory animal procedures. The study protocol received approval from the AnimCare Animal Research Ethics Committee of North-West University, South Africa (Ethics number: NWU-00055-19-S5) and the Animal Research Ethics Committee of the University of Cape Town, South Africa (Ethics number: FHS AEC 019-023).

## Author Contributions

LM headed the project. FH, LM, RD, RB, and AN conceptualized and planned the experiments. FH, LM, MO, and SP investigated and performed the experiments and contributed to the interpretation of the results. FH, LM, and MO analyzed the data. FH took the lead in writing the manuscript. RD, FB, and SP were involved in acquiring resources and funding for the experiment. All authors provided critical feedback and helped to shape the research, analysis, and manuscript, read and approved the final manuscript.

## Conflict of Interest

The authors declare that the research was conducted in the absence of any commercial or financial relationships that could be construed as a potential conflict of interest.

## Publisher's Note

All claims expressed in this article are solely those of the authors and do not necessarily represent those of their affiliated organizations, or those of the publisher, the editors and the reviewers. Any product that may be evaluated in this article, or claim that may be made by its manufacturer, is not guaranteed or endorsed by the publisher.

## Abbreviations

BSL3: Bio-safety hazard level 3
CFU: Colony-forming units
COX-2: Cyclooxygenase-2
DHA: Docosahexaenoic acid
EPA: Eicosapentaenoic acid
FA: Fatty acid
FAD: Fatty acid deficient
FAS: Fatty acid sufficient
GM-CSF: Granulocyte-macrophage colony-stimulating factor
Hb: Hemoglobin
HDHA: Hydroxydocosahexaenoic acid
HEPE: Hydroxyeicosapentaenoic acid
HETE: Hydroxyeicosatetraenoic acid
IFN-γ: Interferon-gamma
IL: Interleukin
LMs: Lipid mediators
MCP-1: Monocyte chemoattractant protein 1
MIP-1α: Macrophage inflammatory protein 1-alpha
Mtb: Mycobacterium tuberculosis
N-3 LCPUFAs: Long-chain polyunsaturated fatty acids
OADC: Oleic acid-albumin-dextrose-catalase
PD1: Protectin D1
PG: Prostaglandin
PUFA: Polyunsaturated fatty acid
PURE: Prospective Urban Rural Epidemiology
RANTES: Regulated on Activation Normal T-cell Expressed and Secreted
RH: Rifampicin and isoniazid
SPMs: Specialized pro-resolving mediators
TB: Tuberculosis
TBXB2: Thromboxane B2
TNF, Tumor necrosis factor TNF-α: Tumor necrosis factor-alpha.
