# Supplementary material for: Adjunct n-3 Long-Chain Polyunsaturated Fatty Acid Treatment in Tuberculosis Reduces Inflammation and Improves Anemia of Infection More in C3HeB/FeJ Mice With Low n-3 Fatty Acid Status Than Sufficient n-3 Fatty Acid Status
Source: Front Nutr. 2021 Aug 24;8:695452. doi: 10.3389/fnut.2021.695452 (PMC8421789; doi:10.3389/fnut.2021.695452)
Supplement: Supplementary file 1 [file Data_Sheet_1.pdf]

Supplementary table and figure

Supplementary table 1: Fatty acid composition of intervention diets

*Diet	Fat source	LA	ALA	DHA	EPA	AA
#EPA/DHA	70 g/kg Soybean oil	3.44 g	0.42 g	0.06 g	0.09 g	< 0.01 g
	27 g/kg Coconut oil			28 % FA	44 % FA	
	3 g/kg Incromega TG4030					
(n-3)FAS	70 g/kg Soybean oil	3.54 g	0.44 g	< 0.01 g	< 0.01 g	< 0.01 g
	30 g/kg Coconut oil					
(n-3)FAD	81 g/kg Coconut oil	1.30 g	0.01 g	< 0.01 g	< 0.01 g	< 0.01 g
	19 g/kg Safflower oil					

*****Per 100g diet**;** ALA, alpha-linolenic acid; LA, linoleic acid; DHA, docosahexaenoic acid; EPA, eicosapentaenoic acid; FA, fatty acid; #EPA/DHA, eicosapentaenoic and docosahexaenoic acid-supplemented diet containing soybean oil at 70 g/kg diet, coconut oil at 27 g/kg diet, Incromega TG4030 oil DHA 500 TG SR (minimum 44% of FA as EPA; minimum 28% of FA as DHA); (n-3) FAS, omega-3 fatty acid-sufficient diet; (n-3) FAD, omega-3 fatty acid-deficient diet.

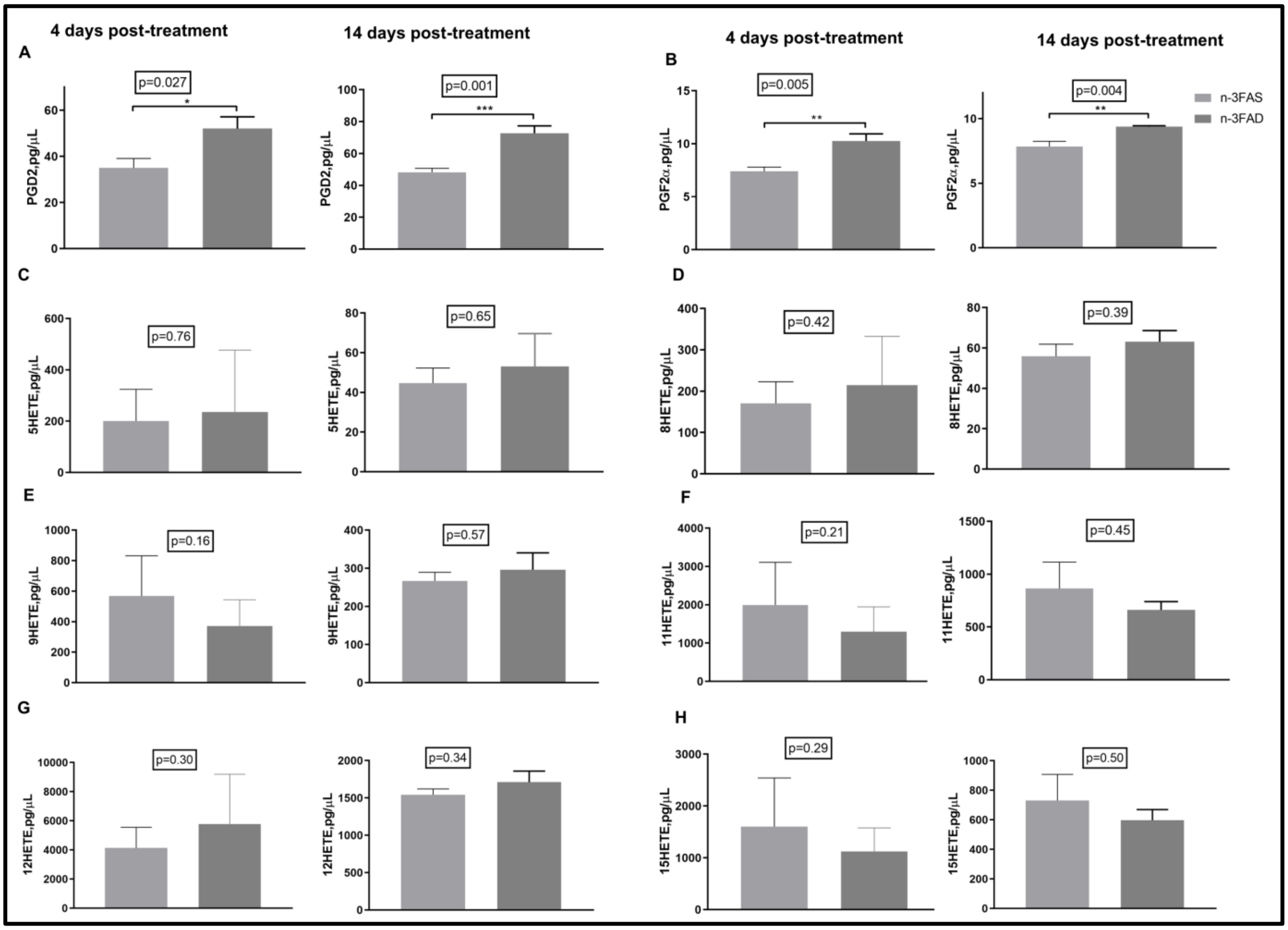

Supplementary figure 1: **Effects adjunct n-3 LCPUFA supplementation to sufficient and low-status n-3 PUFA mice on pro-inflammatory lipid mediators in crude lung homogenates.**

A) PGD2, B) PGF2α, C) 5-HETE, D) 8-HETE, E) 9-HETE, F) 11-HETE, G) 12-HETE and H) 15-HETE. The data are represented as mean ± SEM of n=6 mice/group and representative of two independent experiments. Unpaired two-tailed t-test was used to compare means, significance at $*P < 0.05$, $**P < 0.01$, ***P < 0.001. HETE, hydroxyeicosatetraenoic acid; PGD2, prostaglandin D2; PGF2 α, prostaglandin F2α; n-3FAS/n-3+, omega-3 fatty acid-sufficient switched to DHA/EPA-enriched diet; n-3FAD/n-3+, omega-3 fatty acid-deficient switched to DHA/EPA-enriched diet.
